# Lactate metabolism in clonal plasma cells and its therapeutic implications in multiple myeloma patients with elevated serum LDH levels

**DOI:** 10.1186/s40170-025-00379-1

**Published:** 2025-02-13

**Authors:** Yogesh Chawla, Emilie I. Anderson, Matthew Smith, Sonia Jain, Laura A. Evans, Jadee Neff, Jin Sung Jang, Isas K. Vazquez Rosario, Dragan Jevremovic, Xuan-Mai Petterson, Sinto Sebastian, Rafael Fonseca, Shaji K. Kumar, Taro Hitosugi, Wilson I. Gonsalves

**Affiliations:** 1 Division of Hematology, Mayo Clinic, Rochester, MN United States; 2 Department of Radiation Oncology, Mayo Clinic, Rochester, MN United States; 3 Department of Laboratory Medicine and Pathology, Mayo Clinic, Rochester, MN United States; 4 Metabolomics Core, Mayo Clinic, Rochester, MN United States; 5 Division of Oncology Research, Mayo Clinic, Rochester, MN United States; 6 Division of Hematology and Medical Oncology, Mayo Clinic, Scottsdale, AZ United States; 7 Department of Pathology, Duke Health, Durham, NC United States

**Keywords:** Multiple Myeloma, LDH, Lactate, Energy metabolism, Metabolomic, Monocarboxylate transporters

## Abstract

**Introduction:**

This study aimed to evaluate the metabolic differences between MM cells derived from patients with elevated serum LDH levels and those without elevated serum LDH levels to identify biological differences that could be exploited for therapeutic purposes.

**Methods:**

We performed transcriptome assessments of CD138 + MM cells derived from patients with elevated serum LDH levels compared to those without elevated serum LDH levels and validated the findings in a larger public dataset. Functional metabolic assessments of our findings were performed using a combination of stable isotope resolved metabolomics (SIRM), bioenergetic flux measurement assays, and live cell analysis in human myeloma cell lines and primary MM patient cells.

**Results:**

We identified *SLC16A1*, responsible for the formation of MCT1, a well-defined bi-directional transporter of lactate in and out of a cell with a predilection to importing extracellular lactate, as differentially expressed between the two groups. This finding was functionally confirmed by higher membranous MCT1 protein expression and SIRM on MM cells derived from patients with elevated serum LDH levels compared to those without elevated serum LDH levels. Finally, disrupting lactate transport in and out of CD138 + MM cells was maximally achievable only with dual inhibition of MCT1 and its partner, MCT4, which was preferentially more cytotoxic in MM cells derived from patients with elevated serum levels of LDH.

**Conclusion:**

MCT1 mRNA and protein expression distinguish MM cells derived from patients with elevated serum LDH levels from those without elevated serum LDH levels. However, only dual inhibition of MCT1 and MCT4 can disrupt lactate transport in multiple myeloma (MM) cells, with preferential cytotoxicity in MM cells from patients with high serum LDH levels.

**Supplementary Information:**

The online version contains supplementary material available at 10.1186/s40170-025-00379-1.

## Introduction

Multiple myeloma (MM) is a clinically and molecularly heterogeneous plasma cell malignancy characterized by the uncontrolled proliferation of clonal plasma cells (PCs) in the bone marrow (BM) with occasional extramedullary involvement [1]. Several clinical and laboratory prognostic factors predict the heterogeneous nature of this disease in patients. One such independent prognostic factor is the serum lactate dehydrogenase (LDH) level. Clinically, MM patients with elevated serum LDH levels more commonly have an aggressive disease course, with extramedullary disease and high tumor burden, and their MM cells tend to have high proliferation rates [2, 3]. However, the underlying differences in the intracellular metabolism of MM cells associated with elevated versus non-elevated serum LDH levels have not been elucidated. In this study, we identified that MM cells from patients with elevated serum LDH levels had higher expression of the monocarboxylate transporter, MCT1, that serves as a compensatory and redundant partner to MCT4 in the transport of lactate across the cell membrane. However, only dual inhibition of MCT1 and MCT4 could disrupt lactate transport in MM cells. Furthermore, MM cells from patients with elevated serum LDH levels were more cytotoxically sensitive to disruption of lactate transport across their cell membrane with dual inhibition of MCT1 and MCT4 function compared to MM cells from patients with non-elevated serum LDH levels.

## Results

### Very elevated serum LDH levels in patients with newly diagnosed MM portends inferior survival compared to conventional risk factors

**Fig. 1:**
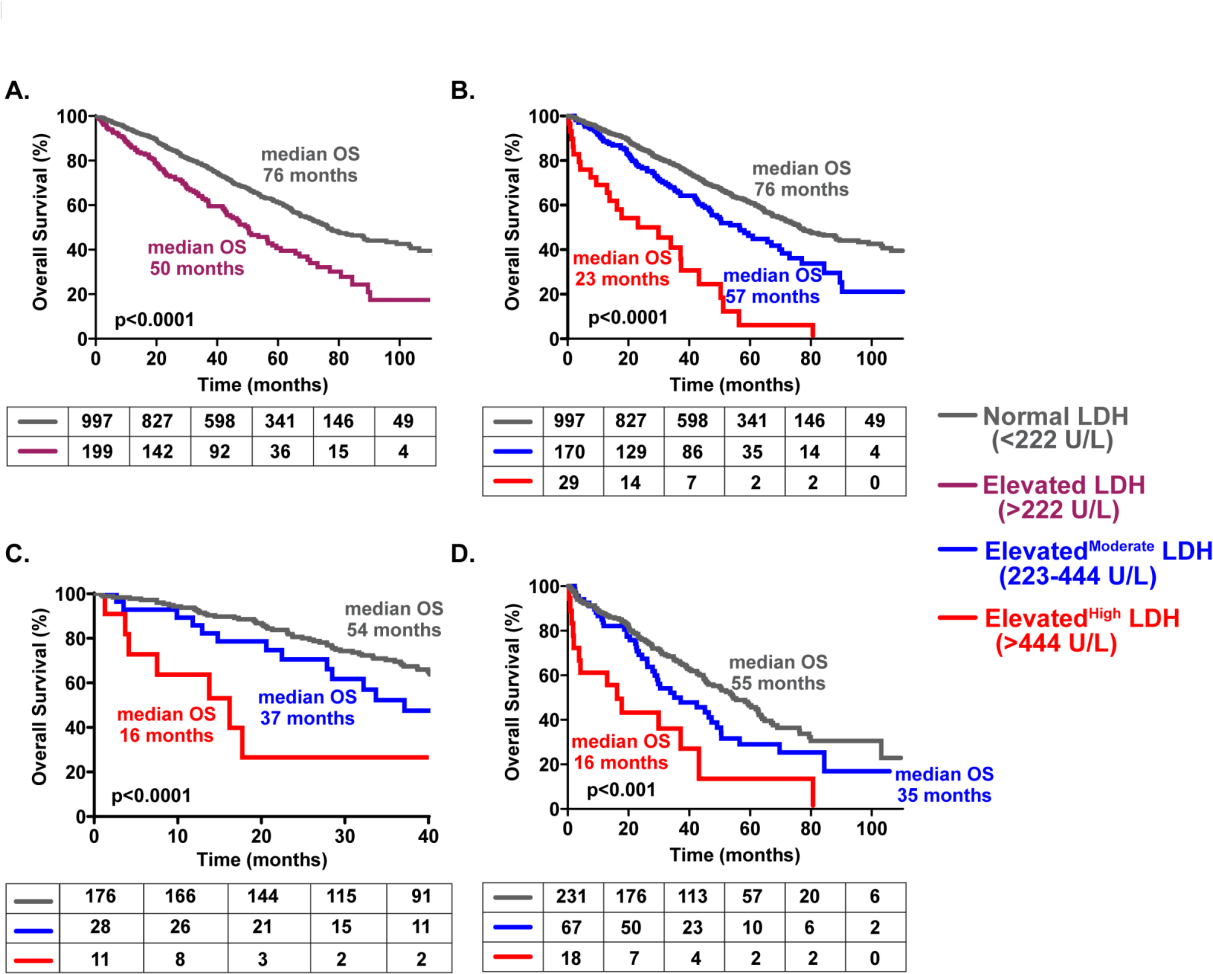
Kaplan Meier curves comparing the overall survival in newly diagnosed MM patients based on (**A**) Normal vs. Elevated serum LDH levels at diagnosis, (**B**) Normal vs. Elevated^Moderate^ vs. Elevated^High^ serum LDH levels at diagnosis, (**C**) Normal vs. Elevated^Moderate^ vs. Elevated^High^ serum LDH levels at diagnosis with high-risk cytogenetics and (**D**) Normal vs. Elevated^Moderate^ vs. Elevated^High^ serum LDH levels at diagnosis with ISS 3 disease

A total of 1,196 consecutive patients diagnosed with MM and who had serum LDH levels available at the time of diagnosis were included in this analysis. The median OS for patients with a normal serum LDH level was 76 months (95% CI: 62–72) compared to 50 months (95% CI: 33–46) in patients with an elevated serum LDH level (Fig. 1A). When further stratified by the level of serum LDH into normal (LDH < 222 U/L; *N* = 997), moderately elevated (Elevated^Moderate^, LDH: 223–444 U/L; *N* = 170), and highly elevated (Elevated^High^, LDH: 445 U/L or greater; *N* = 29), the median OS was 76 months, 57 months and 23 months respectively (*P* < 0.001) (Fig. 1B). Patients with Elevated^High^ levels were more likely to have ISS 3 disease, a high proliferation rate defined by the plasma cell labeling index greater than 3% and a bone marrow plasma cell percentage greater than 50% (Supplementary Table 1). Among patients with high-risk cytogenetics, the median OS was 54 months, 37 months, and 16 months, respectively (*P* < 0.001) for normal, Elevated^Moderate^, and Elevated^High^ LDH levels (Fig. 1C). Finally, the impact of the different LDH levels on OS was analyzed based on ISS stage 3 (available in 1,152 patients), and the median OS was 55 months, 35 months, and 16 months, respectively (*P* < 0.001) for normal, Elevated^Moderate^, and Elevated^High^ LDH levels (Fig. 1D). Together, these observations demonstrate that in patients already expected to have a reduced OS, such as those with high-risk cytogenetics or ISS 3 disease, any elevated serum LDH level at diagnosis and especially those with Elevated^High^ serum LDH levels portend a far worse-than-expected OS outcome.

### Metabolic gene expression differences between primary CD138 + MM cells from patients with elevated serum LDH and non-elevated serum LDH levels

Primary CD138 + MM cells were obtained from the BM aspirates of patients with MM with elevated serum levels of LDH (*N* = 10) (Median LDH: 435 U/L) and patients with MM who had non-elevated serum levels of LDH (*N* = 11) (Median LDH: 165 U/L). The clinical characteristics of these patients are documented in Supplementary Table 2. Bulk cell RNA sequencing of these primary CD138 + MM cells identified 1,363 genes to be significantly higher expressed and 180 genes significantly lower expressed in the elevated LDH group compared to the non-elevated LDH group (Supplementary Fig. 1A).

**Fig. 2:**
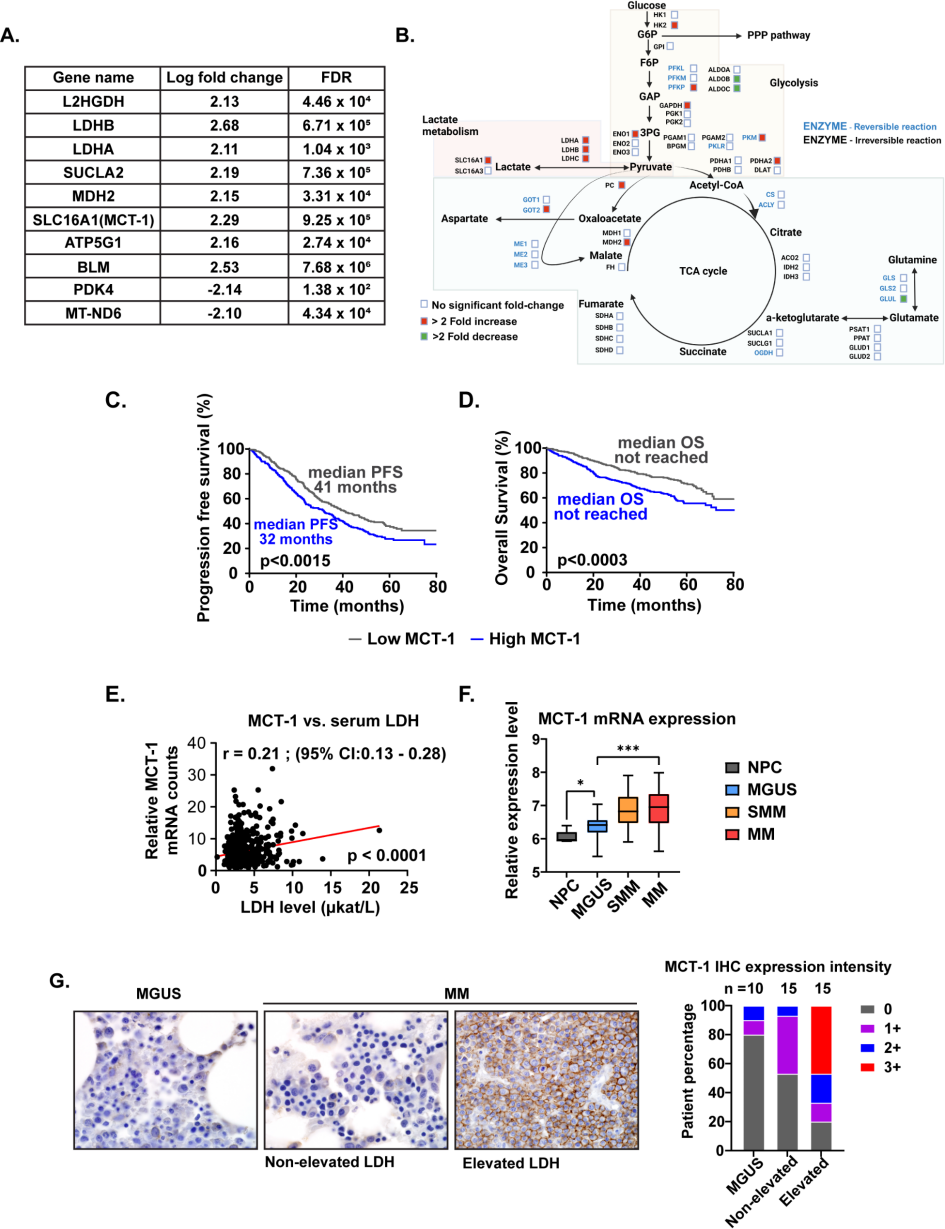
(**A**) Top 10 differentially expressed genes associated with cellular metabolism, (**B**) Differentially expressed genes associated within the glycolysis and TCA cycle metabolic pathways between the MM cells derived from patients with elevated (*N* = 10) vs. non-elevated (*N* = 11) serum LDH levels at the time of sampling. Kaplan Meier curves comparing the (**C**) PFS and (**D**) OS in newly diagnosed MM patients based on high vs. low *MCT1* mRNA expression in the MMRF CoMMpass database. (**E**) Correlation of relative *MCT1* mRNA counts and serum LDH levels at diagnosis in newly diagnosed MM patients in the MMRF CoMMpass database. (**F**) Relative mRNA expression levels of MCT1 in normal plasma cells from healthy donors and CD138 + plasma cells derived from patients with either MGUS, SMM or MM. (**G**) Comparison of MCT1 protein expression on the cell membrane expression of CD138 + plasma cells between patients with MGUS, non-elevated serum LDH levels and elevated serum LDH levels at the time of sampling

To specifically focus on the differences in cellular metabolism between these two groups, the top 10 differentially expressed genes associated with cellular metabolism are depicted in Fig. 2A and are strongly interrelated to each other (Supplementary Fig. 1B) and involved in central carbon energy metabolism (CCEM). Furthermore, a comprehensive assessment of the expression of all major genes responsible for producing key enzymes involved in the glycolysis and TCA cycle metabolic pathways (Fig. 2B) demonstrated that most of the genes associated with enzymes involved in lactate metabolism had more than two log2-fold differentials between the groups. *SLC16A1* was particularly interesting as it had one of the highest fold differences (2.3x) in mRNA. *SLC16A1* leads to the translation of Monocarboxylate transporter-1 (MCT1) which is a bidirectional transporter of lactate in and out of the cell and has been closely associated in function with Monocarboxylate transporter-4 (MCT4).

### Clinical significance of MCT-1 mRNA and protein expression in primary CD138 + MM cells in the spectrum of plasma cell disorders

The clinical significance of *SLC16A1 or MCT1* mRNA expression in CD138 + MM cells was evaluated using the MMRF CoMMpass database. Of 766 patients with NDMM, patients with higher mRNA expression of *MCT1* based on the relative mRNA levels above the median of the entire cohort had a median PFS and OS of 32 months and not reached compared to 41 months and not reached for the low *MCT1* group (PFS: *p* = 0.0015, Fig. 2C and OS: *p* = 0.003, Fig. 2D). Furthermore, patients in the higher mRNA expression of *MCT1* group were more likely to have an elevated serum LDH level at diagnosis than those without a higher mRNA expression of *MCT1* (32% vs. 24%, *p* = 0.035). Serum LDH levels were directly correlated with the *MCT1* mRNA levels within the corresponding CD138 + cells (Fig. 2E). Next, using an existing gene expression dataset, GSE6477, the relative mRNA expression of *MCT1* was significantly increased in MM (*N* = 41) and SMM (*N* = 33) compared to MGUS (*N* = 20), and the latter was higher than the normal PCs obtained from healthy volunteers (*n* = 5) (Fig. 2F). These findings support the possibility of *MCT1* expression being associated with the pathogenesis and progression of MM from MGUS.

To verify if the differential expression of *MCT1* at the mRNA level was borne out in the membrane-based protein expression of MCT1, patients with MGUS (*N* = 10), MM patients with elevated serum LDH levels (*N* = 15), and MM patients with non-elevated serum LDH levels (*N* = 15) had immunohistochemical (IHC) assessments of MCT1 on the primary CD138 + MM cells in their BM core biopsy specimens. A higher percentage of 2 + and 3 + membranous expression of MCT1 was observed on the primary CD138 + MM cells from MM patients with elevated serum LDH levels compared to those with non-elevated serum LDH levels (Fig. 2G). Together, this confirmed the increased MCT1 protein expression in primary CD138 + MM cells from patients with elevated serum LDH levels compared to those with non-elevated serum LDH levels. Furthermore, most primary CD138 + MGUS cells had 0 + or 1 + membranous expression of MCT1, which also supports the earlier stated possibility of *MCT1* expression being associated with the pathogenesis and progression of MM from MGUS.

### Clinical significance of MCT-4 mRNA and protein expression in primary CD138 + MM cells in the spectrum of plasma cell disorders

Since MCT1 is known to function in close collaboration with MCT4 to maintain the intracellular pH, we evaluated the clinical significance of MCT4. In the CoMMpass database, higher mRNA expression of *SLC16A4* or *MCT4* based on the levels above the median expression of the group had a shorter median PFS and OS of 32 months and 72 months compared to 39 months and not reached for the low MCT4 group (PFS: *p* = 0.0731, Supplementary Fig. 1C and OS: *p* = 0.0287, Supplementary Fig. 1D). However, there was no correlation of serum LDH levels with the MCT4 mRNA levels within the corresponding CD138 + cells (Supplementary Fig. 1E). Finally, we evaluated the relative mRNA expression of *SLC16A3* or *MCT4* in the spectrum of plasma cell disorders using the aforementioned GSE6477 dataset. In contrast to MCT1, MCT4 was significantly increased in MGUS compared to SMM, MM, and normal PCs obtained from healthy volunteers (Supplementary Fig. 1F).

### Utilization of extracellular lactate in clonal plasma cells as carbon substrate for the formation of TCA cycle intermediates

**Fig. 3:**
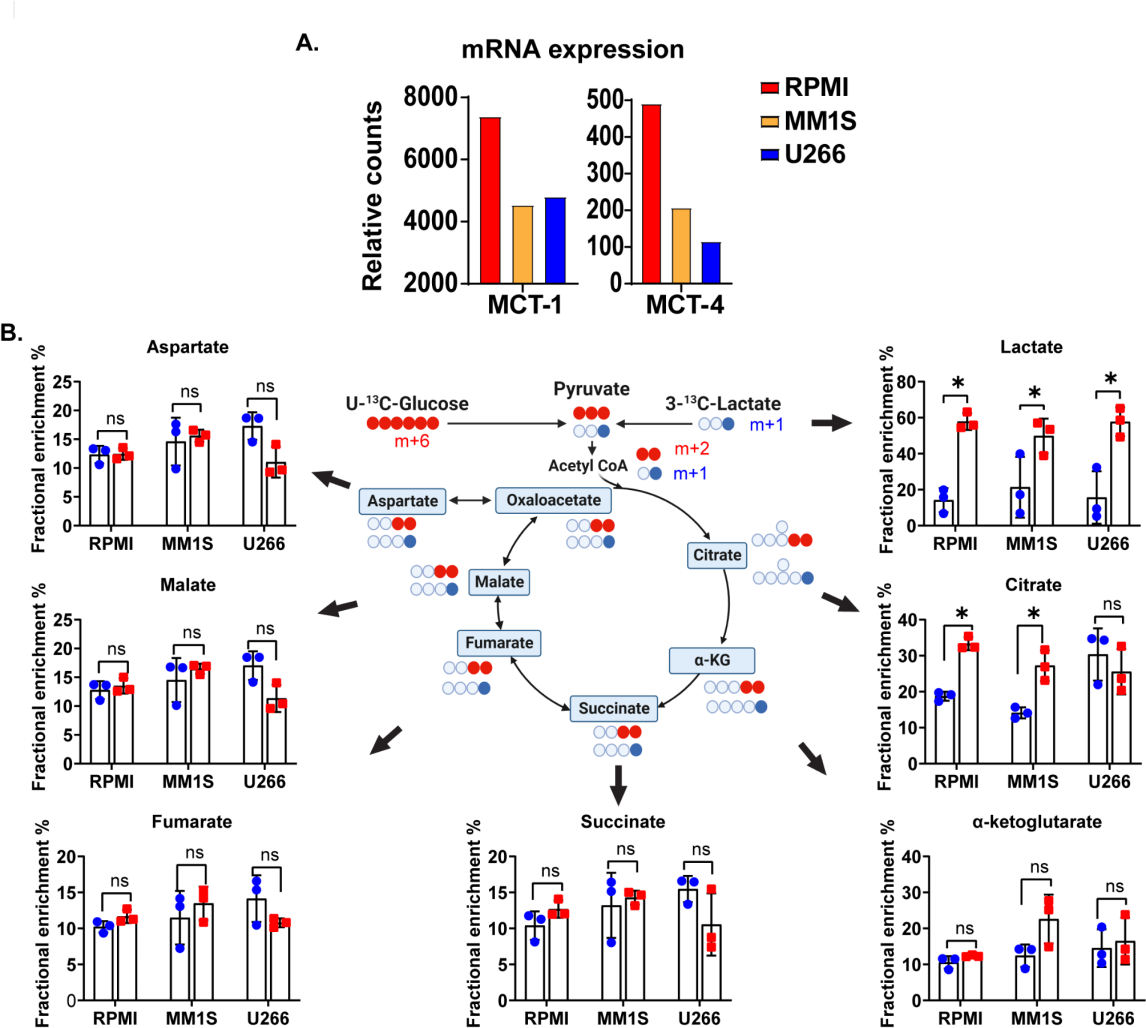
(**A**) Basal mRNA expression of MCT1 and MCT4 in various HMCLs (RPMI-8226, MM1S and U266). (**B**) The expected and observed ^13^C isotopomers with the first turn of the TCA cycle in HMCLs (RPMI-8226, MM1S and U266) cultured in RPMI-1640 cell culture media containing 1mM of 100% enriched 3- ^13^C-Lactate and 100% enriched U-^13^C-Glucose Error bars represent standard error of the mean (SEM). n.s. is non-significant, ^#^*p* < 0.1, *p < 0.05, **p < 0.01, and ***p < 0.001 and ****p < 0.0001 by paired t test

Both MCT1 and MCT4 serve as bidirectional lactate transporters into and out of the cell, but MCT1 preferentially takes up extracellular lactate, and MCT4 preferentially exports out intracellular lactate [4]. Thus, the increased expression of MCT1 on MM cells from the BMs of patients with elevated serum LDH levels suggests that extracellular lactate is likely being taken up by MM cells and not just released out of the cell into the microenvironment. Furthermore, it was recently demonstrated that the uptake of extracellular lactate is a significant contributor of carbon substrate to the TCA cycle that exceeds that of the contribution by extracellular glucose [5]. Given the prognostic relevance of *MCT1* and *MCT4* mRNA expression in NDMM, lactate transport activity across the cell membrane in CD138 + MM cells was evaluated using SIRM-based tracing studies in various human myeloma cell lines (HMCLs) such as RPMI-8226, MM1S, and U266. The baseline mRNA expression levels (Fig. 3A) of MCT1 and MCT4 were evaluated in these three cell lines, confirming the presence of varying levels of MCT1 and MCT4. Furthermore, since the pathogenesis of MM can be dependent on increased MYC protein expression and there was an increase in *MCT1* mRNA expression in MM CD138 + cells compared to MGUS CD138 + cells, an assessment of the correlation of MYC with MCT1 and MCT4 expression was performed using MYCi975, which is a MYC inhibitor that engages MYC intracellularly and disrupts MYC/MAX-dimers that impairs MYC-driven gene expression. In both human myeloma cell lines (HMCLs), RPMI-8226 and MM1S, inhibition of MYC protein expression by MYCi975 was associated with decreased MCT1 expression and a compensatory increase of MCT4 further supporting the association of MCT1 in the pathogenesis of MM (Supplementary Fig. 1G).

Dual stable isotope tracing in cytogenetically different HMCLs (RPMI-8226, MM1S, and U266) was performed in cell culture media containing 1mM (approximate physiologic concentrations in the human body) of 100% enriched 3- ^13^C-Lactate and 100% enriched U-^13^C-Glucose as previously described. The expected and observed isotopomers in the various TCA intermediates are demonstrated in Fig. 3C. With the exception of lactate and citrate, the contribution of carbon substrate derived from extracellular lactate to form the TCA cycle intermediates was similar to those derived from extracellular glucose in all HMCLs. This utilization of extracellular lactate is independent of the mRNA expression of *MCT1* since despite U266 having the lowest *MCT1* and *MCT4* mRNA than RPMI-8226 and MM1S, the degree of extracellular lactate uptake and anaplerosis into the TCA cycle was not different between them. In summary, in addition to just producing lactate and releasing it extracellularly, HMCLs reutilize a significant amount of extracellular lactate in vitro. Counterintuitively, the extent of this utilization of extracellular lactate is independent of *MCT1* or *MCT4* mRNA expression in these HMCLs.

### Contribution of MCT1 and MCT4 in the lactate exchange in and out of MM cells

**Fig. 4:**
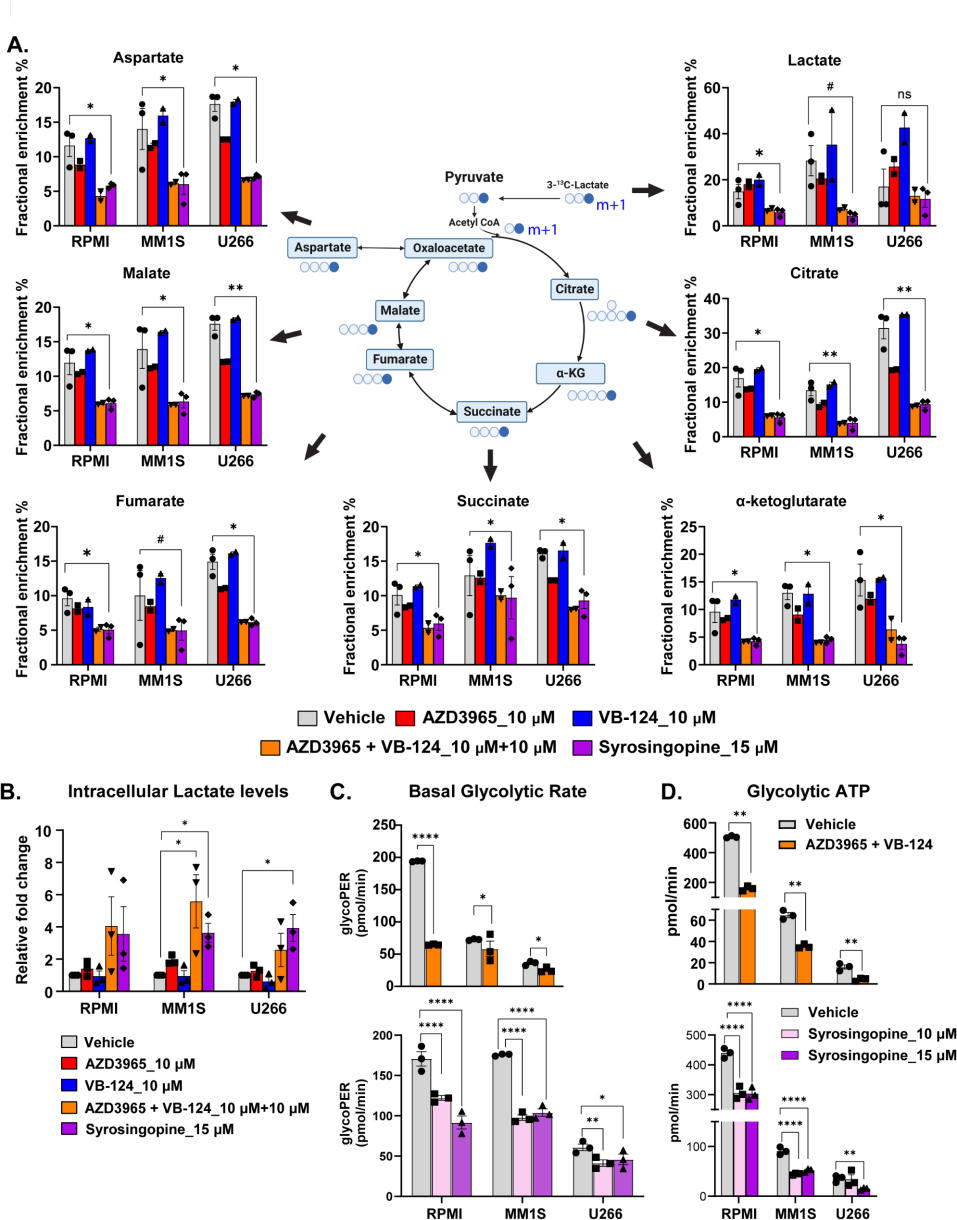
(**A**) Observed (m + 1) lactate fractional enrichment in HMCLs (RPMI-8226, MM1S and U266) cultured in RPMI-1640 cell culture media containing 1mM of 100% enriched 3-^13^C-Lactate and treated with one of five different conditions: DMSO, AZD3965 10µM alone, VB124 10µM alone, combined AZD3965 10µM with VB124 10µM and syrosingopine 15µM. The entire experiment was repeated two times with all five conditions and the third replicate was conducted only with the DMSO and syrosingopine conditions. (**B**) Intracellular levels of lactate in HMCLs (RPMI-8226, MM1S and U266) treated with DMSO, AZD3965 10µM alone, VB124 10µM alone, combined AZD3965 10µM with VB124 10µM and syrosingopine 15µM. (**C**) Basal glycolytic rate and (**D**) Glycolytic ATP production rate of HMCLs (RPMI-8226, MM1S and U266) treated with combined AZD3965 10µM with VB124 10µM and syrosingopine 15µM compared to their respective controls. Error bars represent standard error of the mean (SEM). n.s. is non-significant, ^#^*p* < 0.1, *p < 0.05, **p < 0.01, and ***p < 0.001 and ****p < 0.0001 by paired t test

To help further characterize the contributions of MCT1 and MCT4 in the exchange of lactate in and out of MM cells, the individual and combined ability of small molecule inhibitors, AZD3965 (specific inhibitor of MCT1 only) [6], VB124 (specific inhibitor of MCT4 only) [7] and syrosingopine (dual inhibitor of MCT1 and MCT4 which is a derivative of reserpine and is a repurposed drug used previously as an anti-hypertensive agent) [8] were used to modulate the activity of MCT1 and MCT4 of HMCLs while incubating them in cell culture media containing 1 mM of 3- ^13^C-Lactate and 11 mM of 100% enriched U- ^13^C-Glucose. As observed in Fig. 4A, the most decrease in the amount of extracellular lactate taken up by the HMCLs and subsequent anaplerosis into the TCA cycle to form its various intermediates was observed by dual and simultaneous inhibition of both MCT1 and MCT4 using two pharmacological approaches: (1) a combination of AZD3965 and VB124 and (2) syrosingospine when compared to MCT1 inhibition alone and minimal effect was observed with MCT4 inhibition alone. These observations corresponded to the intracellular lactate concentration being increased with dual and simultaneous MCT1 and MCT4 inhibition compared to mild increase with MCT1 inhibition alone and almost no increase with MCT4 inhibition alone (Fig. 4B). No changes were noted in the lactate levels in the extracellular media (Supplementary Fig. 2A). Together, this data suggests that MCT1 and MCT4 serve as major exporters of intracellular lactate out of the MM cell and can compensate for each other’s loss in function, MCT1 more than MCT4. As a result, only with dual inhibition of MCT1 and MCT4 can the intracellular lactate levels increase, and this limits the entry of extracellular lactate into the MM cell to undergo anaplerosis into the TCA cycle.

The functional effects of dual MCT1 and MCT4 inhibition in HMCLs using the combination of AZD3965 and VB124 or syrosingopine alone was confirmed by observing significant decreases in the basal glycolytic rates (Fig. 4C) and glycolytic ATP production rates (Fig. 4D) upon treatment with the combination of AZD3965 and VB124 as well as with increasing doses of syrosingopine. On the contrary, the TCA cycle isotopomer patterns demonstrated a higher fraction of (m + 4) isotopomer of citrate and (m + 3) isotopomers of the other TCA cycle intermediates reflective of isotopomers generated from a 2nd turn in the TCA cycle with dual MCT1 and MCT4 inhibition using both the combination of AZD3965 and VB124 as well as syrosingopine but not with vehicle control and isolated MCT1 or MCT4 inhibition (Supplementary Fig. 3). This isotopomer data suggests that there is at least a transient increased activity or flux of the TCA cycle as compensation for decreased glycolytic activity and total ATP generation with dual MCT1 and MCT4 inhibition. Finally, since we have previously shown that extracellular glutamine is utilized for the anaplerosis of carbon substrate into the TCA cycle [9, 10], we evaluated if individual or combined inhibition of MCT1 and MCT4 would increase the utilization of extracellular glutamine to support the TCA cycle activity by incubating the different HMCLs in cell culture media containing 2 mM of 100% enriched U-^13^C-Glutamine. There was no increase in glutamine anaplerosis into the TCA cycle observed in any of the different HMCLs but rather some decrease in glutamine anplerosis was noted in RPMI-8226 and U266 cell lines especially with syrosingopine treatment (Supplementary Fig. 4). Together, these data suggest that dual inhibition of MCT1 and MCT4 prevents the export of intracellular lactate extracellularly and concurrently reduces the entry of extracellular lactate back into the cell, as observed by the decrease in its anaplerosis into the TCA cycle. These changes ultimately lead to a decrease in the glycolytic rate of MM cells, with a compensatory increase in the TCA cycle flux utilizing extracellular glucose but not glutamine.

### Dual inhibition of MCT1 and MCT4 with syrosingopine in multiple myeloma

**Fig. 5:**
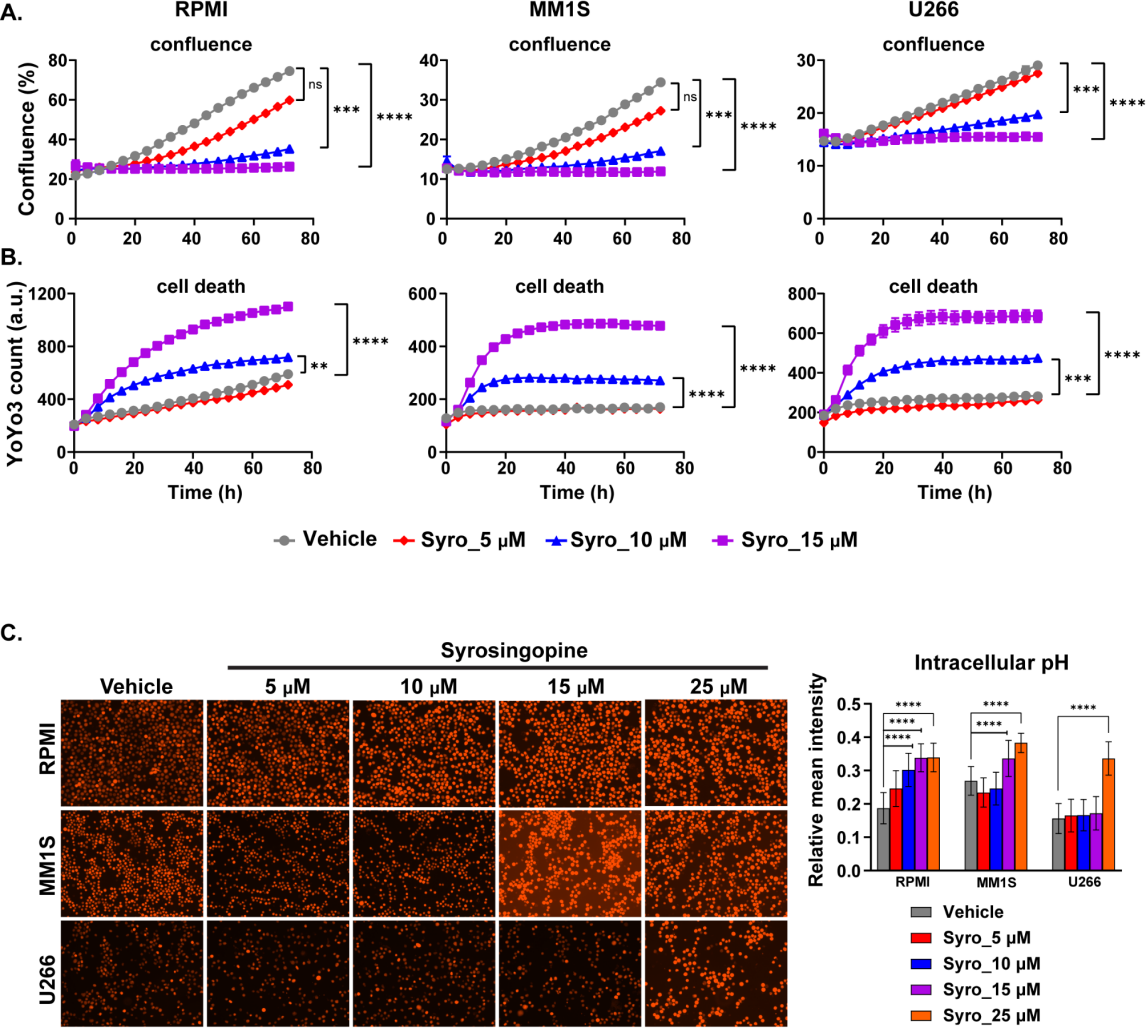
(**A**) Proliferation measured by confluency and (**B**) Cell death measured by YoYo3 fluorescence via live cell imaging in HMCLs (RPMI-8226, MM1S, and U266) treated with DMSO or different dose levels of syrosingopine. (**C**) Relative intracellular pH in HMCLs (RPMI-8226, MM1S, and U266) treated with different dose levels of syrosingopine. All cell culture experiments were repeated three times in each HMCL and one representative replicate experiment is demonstrated. n.s. is non-significant, ^#^*p* < 0.1, *p < 0.05, **p < 0.01, and ***p < 0.001 and ****p < 0.0001 by an independent t test

Since dual MCT1 and MCT4 inhibition was capable of disrupting the transport of lactate in and out of MM cells with a concurrent reduction in their glycolytic rate, the antiproliferative and cytotoxic effects of syrosingopine were evaluated using live cell imaging on a Sartorius Incucyte. There was a statistically significant dose related reduction in the development of confluency of all the different HMCLs (Fig. 5A) and a concurrent increase in the amount of cell death (Fig. 5B) quantified by the amount of YoYo3 dye in the different HMCLs. Interestingly, the decrease in confluency and increase in cell death observed with syrosingopine was not replicated with the combination of AZD3965 and VB124, even at significantly higher doses (20–40 µM of each drug), to account for potential differences in drug potency *(data not shown)*. This suggests that syrosingopine’s cytotoxic effects may involve additional mechanisms warranting further investigation. Nevertheless, to determine whether the increased cytotoxicity observed in HMCLs with dual MCT1 and MCT4 inhibition with syrosingopine was associated with a more acidic intracellular cytoplasm, an assessment of intracellular pH in the various HMCLs was performed after 24 h of treatment with syrosingopine using the pHrodo™ Red AM Intracellular pH Indicator dye assay. The results demonstrated statistically significant decreases in intracellular pH, particularly at the highest concentration of 15 µM syrosingopine, supporting the accumulation of intracellular lactate (Fig. 5C). This was accompanied by a concurrent increase in cytotoxicity and a reduction in proliferation, as shown in Fig. 5A and B. Syrosingopine treatment elicited a dose-dependent decrease in intracellular pH in the RPMI-8226 and MM1S HMCLs. In contrast, in the U266 cell line, a significant reduction in intracellular pH was observed only at the 15 µM concentration.

**Fig. 6:**
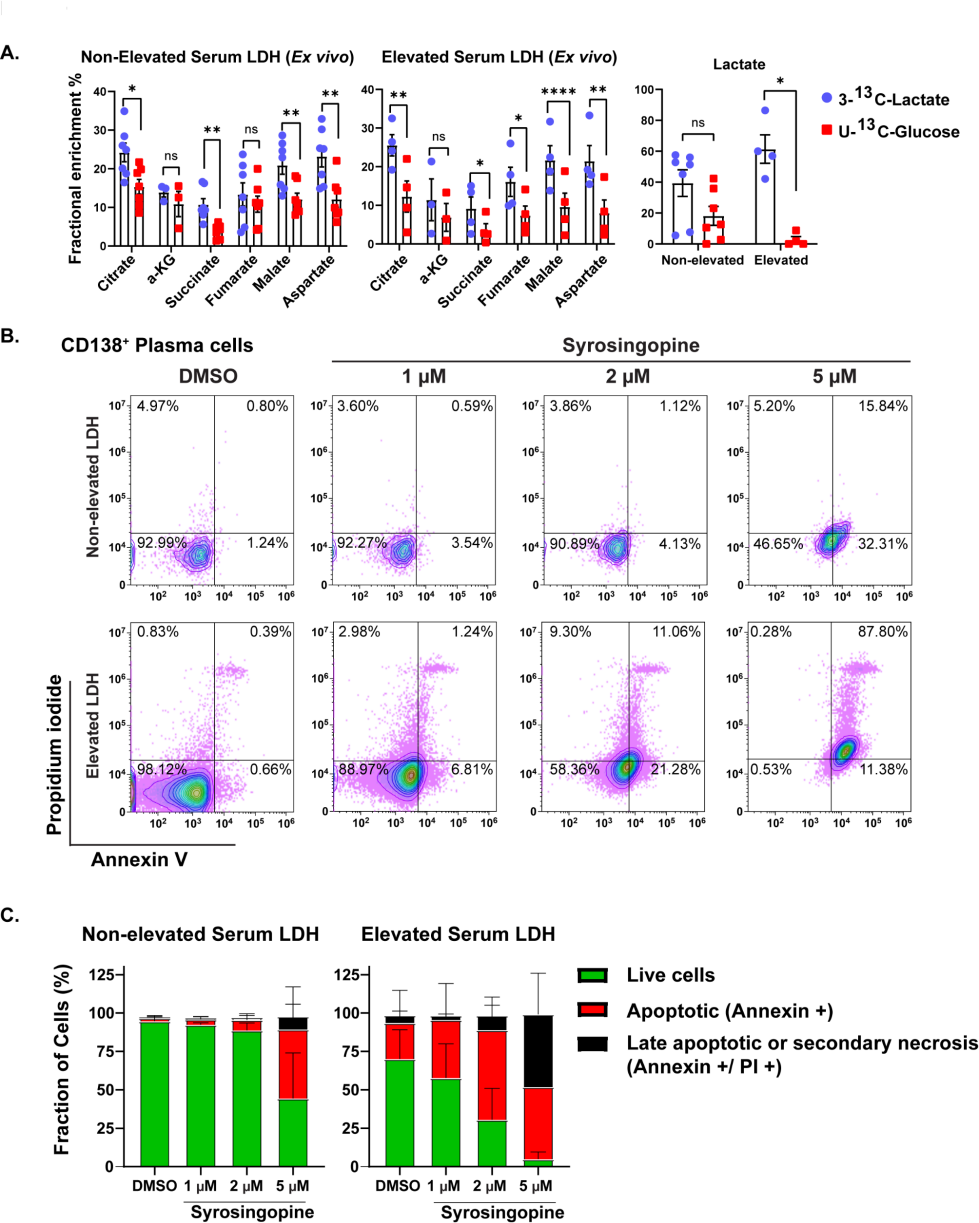
(**A**) The observed ^13^C isotopomers of the TCA cycle intermediates with the first turn of the TCA cycle and intracellular lactate in primary CD138 + MM cells derived from the BM of patients with MM cultured ex vivo in RPMI-1640 cell culture media containing 1mM of 100% enriched 3- ^13^C-Lactate and 100% enriched U- ^13^C-Glucose. Error bars represent standard error of the mean (SEM). n.s. is non-significant, ^#^*p* < 0.1, *p < 0.05, **p < 0.01, and ***p < 0.001 and ****p < 0.0001 by paired t test. (**B**) Individual flow plot examples and (**C**) Summary of 24 h PI/Annexin flow cytometry assessment of ex vivo treatment of primary CD138 + MM cells derived from the bone marrow of patients with either elevated or non-elevated serum LDH levels at the time of sampling

Finally, we sought to determine whether a similar utilization of extracellular lactate uptake is observed in primary MM cells based on their serum LDH levels. Ex vivo culturing of primary CD138 + MM cells derived from the BM of prospective patients with MM with and without elevated serum LDH levels was performed in cell culture media containing 1mM (approximate physiologic concentrations in the human body) of 100% enriched 3- ^13^C-Lactate and 100% enriched U-^13^C-Glucose as previously described. The observed ^13^C isotopomers with the first turn of the TCA cycle are depicted in Fig. 6A. Many TCA cycle intermediates were produced using more carbon substrate derived from extracellular 3-^13^C-lactate than extracellular U-^13^C-glucose. However, there was no difference in the degree of utilization of extracellular 3- ^13^C-lactate to form the different TCA cycle intermediates whether the MM cells were derived from patients with elevated (*N* = 4) or non-elevated (*N* = 7) serum LDH levels at the time of the BM aspiration except for intracellular lactate which was almost entirely derived from extracellular 3- ^13^C-lactate in the CD138 + cells derived from patients with elevated serum LDH levels.

The clinical effects of dual MCT1 and MCT4 inhibition with different doses of syrosingopine were evaluated in primary BM mononuclear cells derived directly and fresh from the BM aspirates of MM patients. Figure 6B provides flow plot examples of index cases demonstrating the increased cytotoxic effects of syrosingopine preferentially in the primary CD138 + MM cells derived from patients with an elevated serum LDH level compared to that from a patient with a non-elevated serum LDH level. Upon flow cytometry assessments 24 h later and based on CD138, PI, and Annexin staining, the primary CD138 + MM cells derived from patients with elevated serum LDH levels (*N* = 3) experienced relatively more late apoptosis/necrosis in a dose-dependent fashion compared to the primary CD138 + MM cells from patients with non-elevated serum LDH levels (*N* = 5) (Fig. 6C). Within each of the primary BM mononuclear cell samples, comparisons of the PI and Annexin staining of primary CD138 + MM cells and their paired CD138- lymphocyte cells demonstrated both higher PI and Annexin staining in the primary CD138 + MM cells after treatment with syrosingopine (Supplementary Fig. 6). Furthermore, the primary CD138 + MM cells from patients with elevated serum LDH levels were more likely to have 2–3 + IHC staining of MCT-1 on their MM cells compared to CD138 + MM cells from patients without elevated serum LDH levels.

## Discussion

LDH is a key enzyme in glycolysis in all cells and is responsible for the interconversion of pyruvate and lactate [11]. This study is the first to demonstrate that a small subset of NDMM patients with very elevated serum LDH levels (Elevated^High^) defined by more than 2 times the upper limit of normal experience have an exceptionally poor OS irrespective of ISS stage and cytogenetic risk. However, prior attempts to assess the biologic differences between the primary CD138 + MM cells from NDMM based on the presence or absence of elevated serum LDH levels using transcriptomics only detected the overexpression of proteolytic and cell adhesion signatures, evasion/suppression of host immune system, hyper-proliferative signatures via cell division and RTK pathways in the primary CD138 + MM cells from patients with elevated serum LDH levels [12]. While this offers a global biological insight into the aggressive nature of the disease in these patients, these large-scale transcriptomic assessments miss capturing specific metabolic differences between the primary CD138 + MM cells in these two groups. Thus, this study is the first to utilize a more focused assessment of the differences in gene expressions associated with CCEM between the primary CD138 + MM cells derived from patients with and without elevated serum LDH levels and, as a result, identified specific genes such as *SLC16A1*, which encodes for the transcellular lactate transporter, MCT1.

Cancer cells have been conventionally characterized to have switched from oxidative phosphorylation to accelerated glycolysis regardless of oxygen availability, resulting in intensive production of lactate (Warburg effect) [13]. To avoid lethal cellular acidosis due to intracellular lactate production, cancer cells upregulate monocarboxylate transporters (MCTs) such as MCT1 and MCT4 that extrude lactate to the tumor microenvironment via a proton-linked mechanism. However, tumors also use lactate as a fuel as extracellular lactate is a significant contributor of carbon substrate to the TCA cycle that exceeds that of the contribution by extracellular glucose [5, 14, 15]. Similarly, in this study, there was significant reutilization of extracellular lactate by primary CD138 + MM cells and HMCLs to support the carbon substrate requirements for forming TCA cycle intermediates. Recent studies highlight a dual role of lactate in the immune tumor microenvironment, distinct from its effects on tumor cells. Lactate contributes to CD8 + T cell exhaustion by upregulating monocarboxylate transporters which leads to increased uptake of lactate and subsequent expression of coinhibitory molecules and reduced functionality [16]. These insights have been leveraged therapeutically [17], as MCT1 inhibition in chimeric antigen receptor (CAR) T cells enhances their cytotoxic activity and antitumoral efficacy [18]. Furthermore, our study also observed that MCT1 expression was implicated in MM tumor progression with mRNA expression levels increasing from benign (MGUS) to malignant disease (MM), which supports prior studies that showed that CD147 expression (the chaperone protein for MCT1) on CD138 + MM cells was regulated by MYC and p53, both of which are key players in MM pathogenesis and prognosis [19–22] and is also associated with levels increasing from MGUS to MM in conjunction with an increased presence of M2 tumor-associated macrophages [23]. Despite the high dependence of multiple myeloma (MM) cells on glutamine—driven by elevated c-Myc expression—and the established link between increased glutamine utilization and MM pathogenesis, the absence of a compensatory rise in glutamine anaplerosis into the TCA cycle following a reduction in extracellular lactate anaplerosis through dual MCT1 and MCT4 blockade remains unresolved. Notably, dual inhibition of MCT1 and MCT4, particularly with the use of syrosingopine in RPMI-8226 and U266 HMCLs, led to a significant decline in glutamine anaplerosis activity. This observation suggests that the anti-proliferative effects of dual MCT1 and MCT4 blockade in MM cells may concurrently suppress their glutamine anaplerosis activity.

Due to the pervasive role of MCT1 in most cancers, MCT1 inhibition serves as a highly attractive therapeutic target with significant pre-clinical activity observed with AZD3965, MCT1 specific inhibitor, both in vitro and in vivo [6, 24]. It causes cell death mainly by necrosis from the accumulation of intracellular lactate and resistance to MCT1 inhibition by AZD3965 has been linked to cells displaying increased MCT4 expression [25]. MCT4 seems to create a compensatory mechanism to allow the lactate export out of the cell to cope with MCT1 blockade. In our study, we observed a similar relationship where only after dual inhibition of MCT1 and MCT4 in the HMCLs with syrosingopine was there dose-dependent cytotoxicity and this was confirmed mechanistically by the increase in intracellular lactate concentrations and decreasing intracellular pH. Hanson et al. also noted a similar cytotoxic effect of complete MCT blockade in MM cells [26]. However, this study uniquely demonstrates that dual MCT1 and MCT4 inhibition with syrosingopine was more potent in primary CD138 + MM cells derived from patients with elevated serum LDH levels than those without elevated serum LDH levels. In addition, the overall selectivity of cytotoxicity was limited to the CD138 + MM cells rather than the non-malignant lymphocytes from the same paired BM aspirate sample. It is important to note that MCT1 has been linked to the formation of a transmembrane complex with the protein CD147 or basigin (BSG) that is regulated by a ubiquitin-independent chaperone-like function of cereblon (CRBN) which is the primary target by which immunomodulatory drugs like thalidomide and lenalidomide mediate their anticancer and teratogenic effects [27]. Furthermore, overexpression of MCT1 was demonstrated to impair the efficacy of lenalidomide in human MM cells, both in vitro and in vivo, and high gene expression levels of SLC16A1 were associated with reduced PFS and OS in MM with lenalidomide-based maintenance therapy [28]. Thus, it is possible that the inferior outcomes observed in NDMM patients with very elevated serum LDH levels could be partly driven by an impaired efficacy of lenalidomide against their MM cells. However, this needs to be further evaluated. In this study, we examined the relationship between basal MCT1 expression and sensitivity to lenalidomide across various HMCLs. Notably, RPMI-8226, which exhibited the highest MCT1 mRNA expression compared to MM1S and U266 (Fig. 3A), showed no significant dose-dependent reduction in proliferation even at 15 µM of lenalidomide, unlike the MM1S and U266 cell lines. (Supplementary Fig. 7).

Several limitations of this study include the selective assessment of only the MCT1/MCT4 axis of lactate transport in MM cells. Other cell membrane transporters and metabolic enzymes may be implicated with lactate exchange across the cell membrane in MM cells, which was beyond the scope of this project but will be explored in the future. While dual pharmacological inhibition of MCT1 and MCT4 appears feasible in vitro with syrosingopine, the concentrations required to observe cytotoxicity are high in the 2–5 micromolar range for primary CD138 + MM cells and 10–15 micromolar range for the HMCLs. Van der Vreken et al. also reported a similar observation with syrosingopine in HMCLs. However, concurrent treatment with metformin increased the cytotoxicity compared to syrosingopine treatment alone [29]. Although small molecule inhibitors with nanomolar potency are more appealing for clinical development, designing more potent and specific inhibitors that replicate the cytotoxic effects of syrosingopine could offer significant therapeutic benefits. Such inhibitors may be particularly advantageous for MM patients with elevated serum LDH levels, who often exhibit more aggressive disease compared to those without elevated LDH levels. Furthermore, it is unclear if there are targets other than MCT1 and MCT4 that syrosingopine is inhibiting, given that the dual treatment combination of AZD3965 and VB124 did not elicit any significant cytotoxicity compared to vehicle controls in the various HMCLs despite these agents being recognized as “tool compounds” in terms of their specificity against MCT1 and MCT4 respectively. Instead, its binding to α-enolase, which catalyzes 2-phosphoglycerate to phosphoenolpyruvate conversion, may contribute to cytotoxicity, though this remains uncertain [30]. Future studies eliciting the possibility of other target proteins on or within MM cells being bound by syrosingopine should be performed.

Nevertheless, this study is the first to selectively focus on the metabolic differences between the CD138 + MM cells derived from patients with elevated serum LDH levels compared to those without elevated serum LDH levels. It brings forth a potential pathway for the development of novel synthetic molecules with more potency to simultaneously inhibit the function of MCT1 and MCT4 or any other novel or pivotal protein being bound by syrosingopine to provide a pharmacologic therapeutic strategy for this very difficult-to-treat MM patient population who continue to experience poor clinical outcomes.

## Methods

### Clinical cohort for assessing the significance of elevated serum LDH levels

Newly diagnosed MM (NDMM) patients seen at the Mayo Clinic, Rochester, between January 1, 2003, and December 31, 2010, within 90 days of their diagnosis were included in this study cohort. Approval for this study was obtained from the Mayo Clinic IRB in accordance with the federal regulations and the principles of the Declaration of Helsinki. The upper limit level of the instituitional reference range for the serum lactate dehydrogenase (LDH) assay was 222 U/L. Thus, any level greater than 222 U/L was considered elevated. Furthermore, among those serum LDH levels greater than 222 U/L but no higher than 444 U/L for serum LDH were considered Elevated^Moderate^, and those levels greater than 444 U/L were considered Elevated^High^. The 444 U/L cutoff was used as this was two times the upper limit of normal. Patients who had a FISH analysis performed on their plasma cells were categorized as having high-risk disease if any of the following abnormalities: t(4;14), t(14;16), or t(14;20) were present at any time during their disease course, or a del 17p within 30 days of the diagnosis or any time before the diagnosis [31, 32]. 

### Transcriptomic assessment of MM cells based on serum LDH levels

For primary CD138 + MM cells and human myeloma cell lines (HMCLs), the total RNA was extracted and evaluated using a Qubit (Thermo Fisher Scientific, MA, USA) and Agilent 2100 Bioanalyzer (Agilent Technologies, Santa Clara, CA, USA), respectively. The TrueSeq RNA Exome Kit (Illumina, CA, USA) generated the mRNA-seq library according to the manufacturer’s protocol. Constructed libraries were quantified by Bioanalyzer 2100 system using the D1000 kit (Agilent) and Qubit dsDNA BR Assay kits (Thermo Fisher Scientific). DESeq2 package [33] was used to normalize data and determine differential expression of RNA-seq between the groups. The false discovery rate (FDR) by Benjamini and Hochberg method was used to adjust for comparison. FDR value less than 0.05 and log 2-fold change ± 2 at raw *p-value* less than 0.05 were considered significant. DE genes related to the interested GO term “cell metabolism” (GO:0044237) were extracted from the Ensembl database through the biomaRt package (biomaRt, RRID: SCR_019214). Gene features with a log2 - fold change larger than 2 and a false discovery rate (FDR) < 0.05 were considered significantly differentially expressed between the two groups.

### Immunohistochemistry assessments of MCT1

The MCT1 immunohistochemical (IHC) stain was performed at the Pathology Research Core (Mayo Clinic, Rochester, MN) using the Leica Bond RX stainer (Leica). FFPE tissues were sectioned at 5 microns and IHC staining was performed on-line. Slides for MCT1 (SLC16A1) stain were retrieved for 10 min using Epitope Retrieval 2 (EDTA; Leica) and incubated in Protein Block (Dako) for 5 min. The MCT1 (SLC16A1) primary antibody (Polyclonal Rabbit; Sigma-Aldrich Catalog#: HPA003324) was diluted to 1:300 in Background Reducing Diluent (Dako) and incubated for 15 min. The detection system used was Polymer Refine Detection System (Leica). This system includes the hydrogen peroxidase block, post primary and polymer reagent, DAB, and Hematoxylin. Immunostaining visualization was achieved by incubating slides 10 min in DAB and DAB buffer (1:19 mixture) from the Bond Polymer Refine Detection System. To this point, slides were rinsed between steps with 1X Bond Wash Buffer (Leica). Slides were counterstained for five minutes using Schmidt hematoxylin and molecular biology grade water (1:1 mixture), followed by several rinses in 1X Bond wash buffer and distilled water, this is not the hematoxylin provided with the Refine kit. Once the immunochemistry process was completed, slides were removed from the stainer and rinsed in tap water for five minutes. Slides were dehydrated in increasing concentrations of ethyl alcohol and cleared in 3 changes of xylene prior to permanent coverslipping in xylene-based medium.The intensity of membranous staining of MCT1 on the CD138 + MM cells were graded by an independent hematopathologist reviewer (J. Neff) and reviewed for concurrence by a second hematopathologist reviewer (D. Jevremovic).

### Small molecule inhibitors of MCT1 and MCT4

The small molecule inhibitors of MCT1 (AZD3965), MCT4 (VB124), dual MCT1/MCT4 (Syrosingopine) were purchased commercially from SelleckChem (www.selleckchem.com). Dimethyl-2-oxoglutarate (Catalog No: 349631) was purchased commercially from Millipore Sigma (www.sigmaaldrich.com).

### Ex vivo _13_C labeling of CD138^+^ MM cells

The freshly obtained BM aspirates from patients underwent Ficoll-Paque gradient separation and the remnant cellular component of the BM aspirate underwent red cell lysis using ACK lysis buffer. The CD138 + MM cells were extracted using positive selection by mixing the cells with a CD138^+^ selection cocktail and anti-CD138 magnetic-activated cell separation microbeads (RoboSep cell separation system, StemCell Technologies Inc.) in an automated RoboSep cell separation system. Purity of the sorted MM cells was confirmed via light chain restriction using slide-based immunofluorescent method. The CD138^+^ MM cells were incubated in RPMI-1640 medium containing 11 mM of U[^13^C^6^] glucose and supplemented with 10% fetal bovine serum (FBS), 2 mM glutamine, and 1 mM of 3- _13_C-lactate. Similar labelling strategy was used for the HMCLs. Both U [_13_C_6_]glucose and 3- _13_C-lactate were purchased from Cambridge Isotope Laboratories.

### Intracellular lactate and TCA cycle intermediates isotopomer analysis

When evaluating the effect of the small molecule inhibitors of MCT1 and/or MCT4 on the in vitro ^13^C labeling of HMCLs, they were first treated for 30 min prior to resuspending them in the fresh cell culture media containing the different ^13^C isotopes. At the time of harvest, the HMCL cell pellets and the primary CD138 + MM cells were resuspended in 50 µl of 1× PBS and sonicated briefly to prepare cell pellet homogenates. A mixture of methanol/acetonitrile (50:50) was added to the homogenates to deproteinate the samples, followed by centrifugation at 19,000 *g* for 10 min. Supernatants were dried under a stream of nitrogen gas. The remaining pellet was saved to perform protein assay. Prepared extracts of HMCLs and spent media were derivatized using ethoxyamine hydrochloride solution in pyridine and subsequently silylated with MTBSTFA (N-Methyl-N-[tert-butyldimethylsilyl] trifluoroacetamide) and 1% tBDMCS (tertbutyldimetheylchlorosilane) (Regis Technologies), followed by overnight incubation at room temperature. After evaporation to dryness, the residues were redissolved in n-decane.

Isotopomer analysis of the intracellular TCA cycle and lactate metabolites from the HMCL cell pellets was performed using an Agilent Technologies 5975 C gas chromatography-mass spectrometry (GC-MS). The MS was operated under electron impact (EI) conditions with selected ion monitoring (SIM). Data were processed using MassHunter quantitative analysis software version B.05.01 build 5.1.315.0 (Agilent Technologies Inc.) for integration of peaks and calculation isotopic ratios. SIM was used to monitor the mole percent enrichment for each analyte, such as the fragment (M0) and all labeled mass isotopomer positions (M1, M2, M3, etc.) up to m + 2 above the number of carbons in the molecule backbone. M/z values of M0 were monitored for the following intermediates: lactate (m/z 261.2), fumarate (m/z 287.1), succinate (m/z 289.1), α-ketoglutarate (m/z 360.2), malate (m/z 419.3), citrate (m/z 591.4), and glutamate (m/z 432.2). The mass isotopomer distribution of each compound was then corrected for natural abundance using the respective standards. An appropriate set of linear simultaneous equations were usesd to calculate mole percent enrichment of TCA cycle intermediates to understand glucose-dependent or lactate-dependent metabolism in the MM cells.

### Proliferation and cytotoxicity assessment assays (in Vitro and Ex vivo)

Cell confluency and death of the various HMCLs treated with different small molecule inhibitors such as AZD3965, VB124 and syrosingopine were measured using an IncuCyte S3 Live-Cell analysis system (Sartorius, Germany). HMCLs were plated in standard 96-well plates coated with poly-L-lysine given the suspension nature of the cells. Images were captured every 4 h up to 72 h from the addition of the vehicle or small molecule of interest in addition to the YoYo3 red fluorescent dye (a measure of cell death). Data was analyzed using the Incucyte ZOOM software, version 2018 A (Sartorius). All experiments were performed in triplicate.

An Annexin/ PI flow cytometry assay was used to measure viability, apoptosis, and necrosis of fresh primary BM mononuclear cells treated with or without drug. A minimum of 2,000,000 cells per tube were washed with Cell Staining Buffer, spun at 400 g for 5 min, and resuspended in 100uL of Annexin Binding Buffer. The cells were stained with Annexin APC and CD138 FITC for 25 min in darkness before being washed again. The cell pellets were resuspended in Annexin binding buffer and stained with 2uL of propidium iodine (PI). The samples were run on a BD Accuri flow cytometer (BD Biosciences). A range of 500,000–1,000,000 events were collected, and plasma cells were identified by their characteristic CD138 bright staining pattern.

### Glycolytic and ATP rate measurement assays

The Agilent Seahorse XF Real-Time ATP Rate Assay Kit (Agilent, Cat. No.: 103592–100) and the XF Real-Time Glycolytic Rate Assay Kit (Agilent, Cat. No.: 103346–100) was used to detect the ATP production rates of mitochondrial oxidative phosphorylation and glycolysis and the glyolytic rate respectively on the Agilent’s Seahorse Bioscience XFp Extracellular Flux Analyzer (Agilent Technologies) according to the manufacturer’s instructions and protocols (Seahorse Bioscience, North Billerica, MA, USA). These assays included the use of a mix of rotenone and antimycin A and oligomycin (ATP rate assay) and 2-deoxyglucose (Glycolytic rate assay) which were added according to the manufacturer’s instructions and protocols (Seahorse Bioscience, North Billerica, MA, USA).

### Intracellular pH assessments

HMCLs treated with vehicle control and various doses of syrosingopine for 12 h in a 6-well plate seeded at a concentration of 1 × 10^6 cells/mL prior to assessment of their intracellular pH via the the pHrodo™ Red AM Intracellular pH Indicator dye (Excitation/Emmission: 560/585) (Catalog number: P35372). A total of 10 µL of pHrodo™ AM Ester was added to 100 µL of PowerLoad™ concentrate (provided with the kit). This cocktail was then diluted into 10 mL of RPMI-1640 media without phenol red at a pH 7.4 to make a staining solution. The growth medium was removed from the cells and wash once with RPMI-1640 media without phenol red which was then replaced with staining solution and incubated at room temperature for 30 min. A second wash was performed to remove the staining solution and the cells were resuspended in RPMI-1640 media without phenol red. Each of the treated cells were imaged on the EVOS M7000 imaging system (ThermoFisher Scientific, Waltham, MA) equipped with a RFP filter (excitation: 542/20 nm, emission: 593/40 nm) and the mean fluorescent intensity captured on individual images were analyzed using the CellPofiler™ cell image analysis software (www.cellprofiler.org).

### Western blot assessments

HMCLs were harvested and lysed to create protein lysates whose concentrations were measured using BCA assay (Pierce, Rockford, IL). Equal amounts of protein were loaded on 12% Tris-Glycine gels and transferred onto nitrocellulose membranes. Membranes were probed with anti-c-MYC (Cell signaling Technology Cat#: 18583 S), anti-MCT1 (Santa Cruz Biotechnology Cat#: SC365501) and anti-MCT4 (Santa Cruz Biotechnology Cat#: SC376140) antibodies (Santa Cruz Biotechnology, Heidelburg, Germany). Blots were stripped and reprobed with anti-GAPDH (14C10) antibody (Cell signaling Technology Cat#: 2118 S) (Cell signaling Technology, Danvers, MA) as a control.

### Statistical analysis

The Fisher’s exact test was used to assess for differences in nominal variables. Differences in continuous variables were compared using the Wilcoxon signed rank test. Cox proportional hazard analysis was used to identify prognostic factors for OS. Survival curves were constructed using the Kaplan-Meier method and compared using the log-rank test. All analyses were performed using JMP 16.0 (Statistical Analysis System (SAS) Institute Inc., Cary, NC, USA).

## Electronic supplementary material

Below is the link to the electronic supplementary material.

Supplementary Material 1

Supplementary Fig. 1: (A) Heatmap reflecting the difference in gene expression between the MM cells derived from patients with elevated (*N* = 10) vs. non-elevated (*N* = 11) serum LDH levels, (B) Interrelationship between the top 10 differentially expressed genes associated with cellular metabolism between the MM cells derived from patients with elevated (*N* = 10) vs. non-elevated (*N* = 11) serum LDH levels. Kaplan Meir curves comparing the (C) PFS and (D) OS in newly diagnosed MM patients based on high vs. low *MCT4* mRNA expression in the MMRF CoMMpass database. (E) Correlation of relative *MCT4* mRNA counts and serum LDH levels at diagnosis in newly diagnosed MM patients in the MMRF CoMMpass database. (F) Relative mRNA expression levels of MCT4 in normal plasma cells from healthy donors and CD138 + plasma cells derived from patients with either MGUS, SMM or MM. (G) Western blots and density bar graphs for MCT1 and MCT4 protein expression at 24 and 48 h upon c-Myc inhibition by 10µM of Myci975 in RPMI and MM1S HMCLs. Error bars represent standard error of the mean (SEM). n.s. is non-significant, ^#^*p* < 0.1, *p < 0.05, **p < 0.01, and ***p < 0.001 and ****p < 0.0001 by an independent t test

Supplementary Fig. 2: Extracellular lactate levels in HMCLs (RPMI-8226, MM1S and U266) treated with DMSO, AZD3965 alone, VB124 alone, combined AZD3965 with VB124 and syrosingopine. Error bars represent standard error of the mean (SEM). n.s. is non-significant, ^#^*p* < 0.1, *p < 0.05, **p < 0.01, and ***p < 0.001 and ****p < 0.0001 by a paired t test

Supplementary Fig. 3: Observed fractional enrichment of the isotopomers of the TCA cycle intermediates in HMCLs (RPMI-8226, MM1S and U266) upon the 2nd turn of the TCA cycle when cultured in RPMI-1640 cell culture media containing 100% enriched U- ^13^C-Glucose and treated with one of five different conditions: DMSO, AZD3965 10µM alone, VB124 10µM alone, combined AZD3965 10µM with VB124 10µM and syrosingopine 15µM. The entire experiment was repeated two times with all five conditions and the third replicate was conducted only with the DMSO and syrosingopine conditions. Error bars represent standard error of the mean (SEM). n.s. is non-significant, ^#^*p* < 0.1, *p < 0.05, **p < 0.01, and ***p < 0.001 and ****p < 0.0001 by a paired t test

Supplementary Fig. 4: Observed fractional enrichment of the isotopomers of the TCA cycle intermediates in HMCLs (RPMI-8226, MM1S and U266) cultured in RPMI-1640 cell culture media containing 2mM of 100% enriched U- ^13^C-Glutamine and treated with DMSO, AZD3965 10µM alone, VB124 10µM alone, combined AZD3965 10µM with VB124 10µM and syrosingopine 15µM. Error bars represent standard error of the mean (SEM). n.s. is non-significant, ^#^*p* < 0.1, *p < 0.05, **p < 0.01, and ***p < 0.001 and ****p < 0.0001 by a paired t test

Supplementary Fig. 5: (A) Proliferation measured by confluency and (B) Cell death measured by YoYo3 fluorescence via live cell imaging in HMCLs (RPMI-8226, MM1S, and U266) treated with DMSO or the combination of AZD3965 (10 µM) and VB124 (10 µM) or syrosingopine (15 µM). (C) Proliferation measured by confluency and (D) Cell death measured by YoYo3 fluorescence via live cell imaging in HMCLs (RPMI-8226, MM1S, and U266) treated with DMSO or AZD3965 (10 µM) alone or VB124 (10 µM) alone or the combination of AZD3965 (10 µM) or VB124 (10 µM). n.s. is non-significant, ^#^*p* < 0.1, *p < 0.05, **p < 0.01, and ***p < 0.001 and ****p < 0.0001 by an independent t test

Supplementary Fig. 6: Comparisons of PI and Annexin staining of primary CD138 + MM cells and their paired CD138- lymphocyte cells categorized by the level of their serum LDH at the time of sampling after after treatment with syrosingopine

Supplementary Fig. 7: Proliferation measured by confluency via live cell imaging in HMCLs (RPMI-8226, MM1S, and U266) treated with DMSO or different lenalidomide dose levels (0.1 µM, 1 µM, 5 µM, 10 µM and 15 µM). n.s. is non-significant, ^#^*p* < 0.1, *p < 0.05, **p < 0.01, and ***p < 0.001 and ****p < 0.0001 by an independent t test

## Data Availability

No datasets were generated or analysed during the current study.
