# Supplementary material for: Lactate metabolism in clonal plasma cells and its therapeutic implications in multiple myeloma patients with elevated serum LDH levels
Source: Cancer Metab. 2025 Feb 13;13:9. doi: 10.1186/s40170-025-00379-1 (PMC11827136; doi:10.1186/s40170-025-00379-1)
Supplement: Supplementary file 1 — Supplementary Material 1 [file 40170_2025_379_MOESM1_ESM.docx]

|  | **Group 1**  **(LDH < 222)** | **Group 2**  **(LDH 223 - 444)** | **Group 3**  **(LDH > 445)** | **p- value** |
| --- | --- | --- | --- | --- |
| **PCLI > 3%** | 7% | 11% | **44%** | **< 0.001** |
| **Age < 70** | 68% | 66% | 62% | 0.729 |
| **ISS 3** | 25% | 47% | **69%** | **< 0.001** |
| **HR FISH** | 9% | 11% | 20% | 0.185 |
| **BMPC > 50%** | 46% | 62% | **87%** | **< 0.001** |

**Supplementary Table 1:** Clinical characteristics of patients based on the serum LDH levels at diagnosis

**Supplementary Table 2:** Clinical characteristics of patients whose CD138+ MM cells were utilized for transcriptomic assessments.

| **Variables** | **Non-Elevated LDH (N = 11)** | **Elevated LDH (N = 10)** |
| --- | --- | --- |
| Median LDH (Range) | 165 (90-181) | 435 (293 – 1388) |
| Median S-phase | 0.6 (0 – 0.8) | 7 (2.1 – 22.7) |
| Time since diagnosis | 5 (0 – 54) | 7.5 (0 – 69) |
| Median OS | 103 months | 7 months |
