# Supplementary figures and images for: Lactate metabolism in clonal plasma cells and its therapeutic implications in multiple myeloma patients with elevated serum LDH levels
Source: Cancer Metab. 2025 Feb 13;13:9. doi: 10.1186/s40170-025-00379-1 (PMC11827136; doi:10.1186/s40170-025-00379-1)

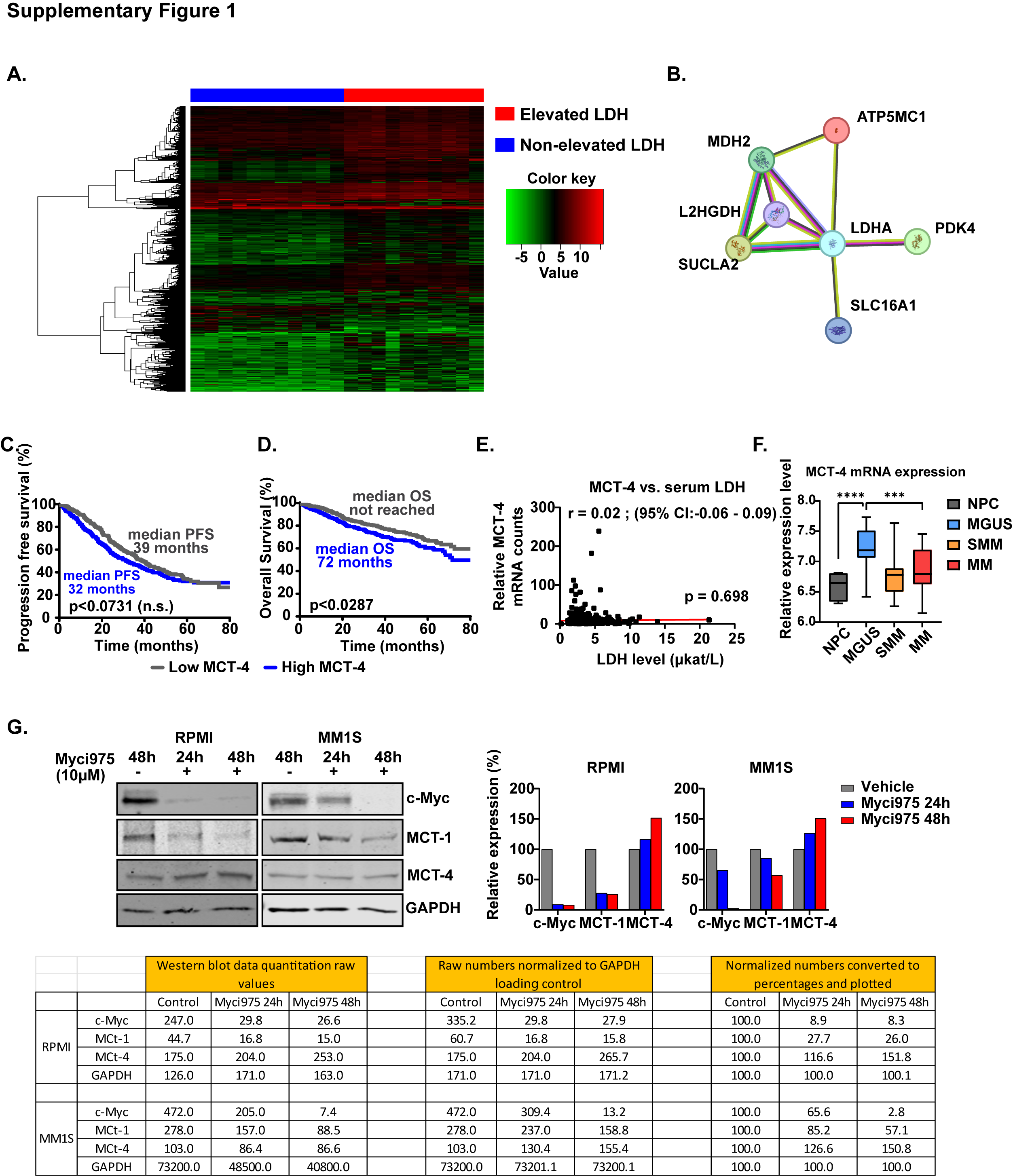

Supplement: Supplementary file 2 — Supplementary Fig. 1: (A) Heatmap reflecting the difference in gene expression between the MM cells derived from patients with elevated (N = 10) vs. non-elevated (N = 11) serum LDH levels, (B) Interrelationship between the top 10 differentially expressed genes associated with cellular metabolism between the MM cells derived from patients with elevated (N = 10) vs. non-elevated (N = 11) serum LDH levels. Kaplan Meir curves comparing the (C) PFS and (D) OS in newly diagnosed MM patients based on high vs. low MCT4 mRNA expression in the MMRF CoMMpass database. (E) Correlation of relative MCT4 mRNA counts and serum LDH levels at diagnosis in newly diagnosed MM patients in the MMRF CoMMpass database. (F) Relative mRNA expression levels of MCT4 in normal plasma cells from healthy donors and CD138 + plasma cells derived from patients with either MGUS, SMM or MM. (G) Western blots and density bar graphs for MCT1 and MCT4 protein expression at 24 and 48 h upon c-Myc inhibition by 10µM of Myci975 in RPMI and MM1S HMCLs. Error bars represent standard error of the mean (SEM). n.s. is non-significant, #p < 0.1, *p < 0.05, **p < 0.01, and ***p < 0.001 and ****p < 0.0001 by an independent t test [file 40170_2025_379_MOESM2_ESM.tif]

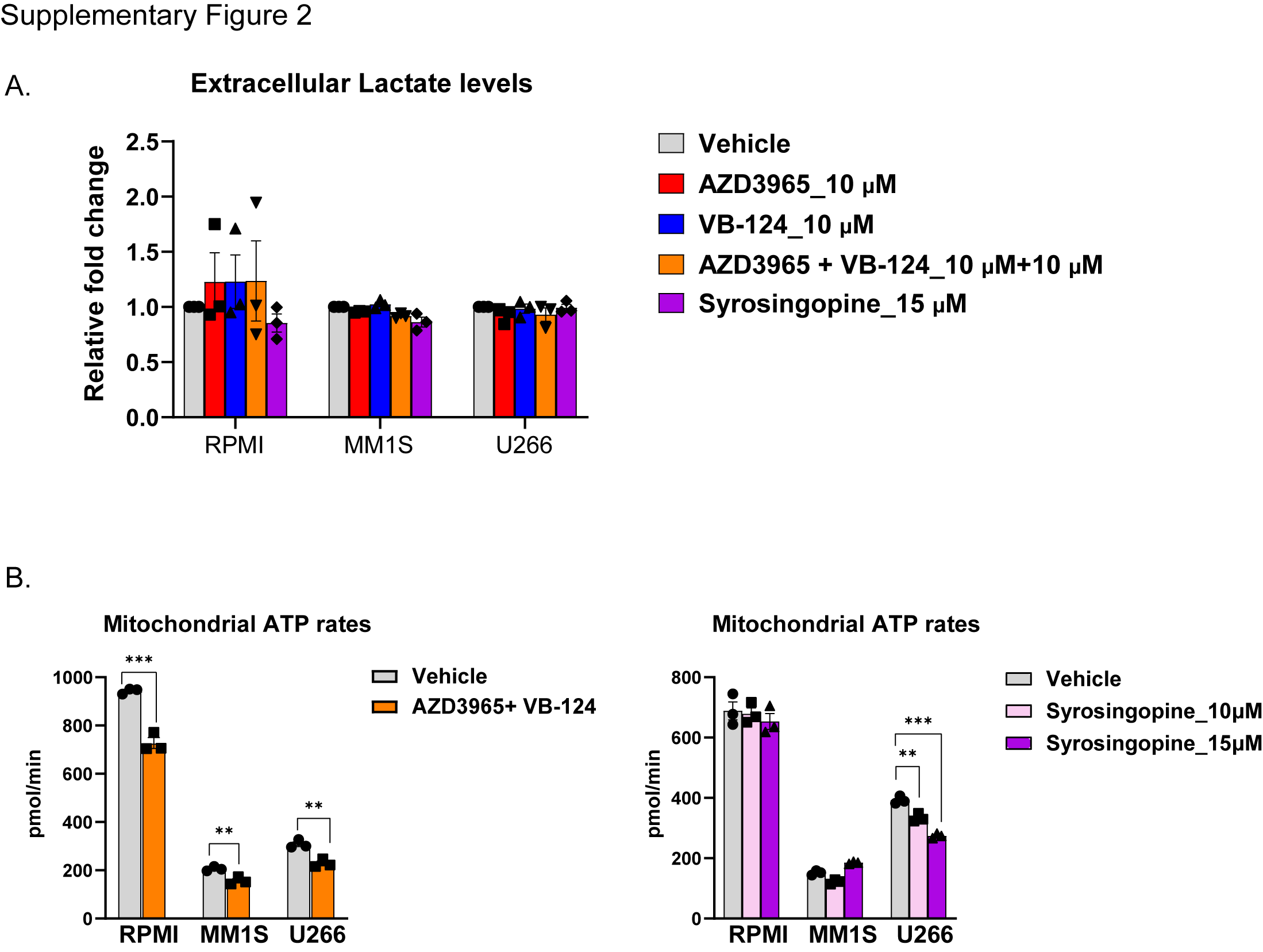

Supplement: Supplementary file 3 — Supplementary Fig. 2: Extracellular lactate levels in HMCLs (RPMI-8226, MM1S and U266) treated with DMSO, AZD3965 alone, VB124 alone, combined AZD3965 with VB124 and syrosingopine. Error bars represent standard error of the mean (SEM). n.s. is non-significant, #p < 0.1, *p < 0.05, **p < 0.01, and ***p < 0.001 and ****p < 0.0001 by a paired t test [file 40170_2025_379_MOESM3_ESM.tif]

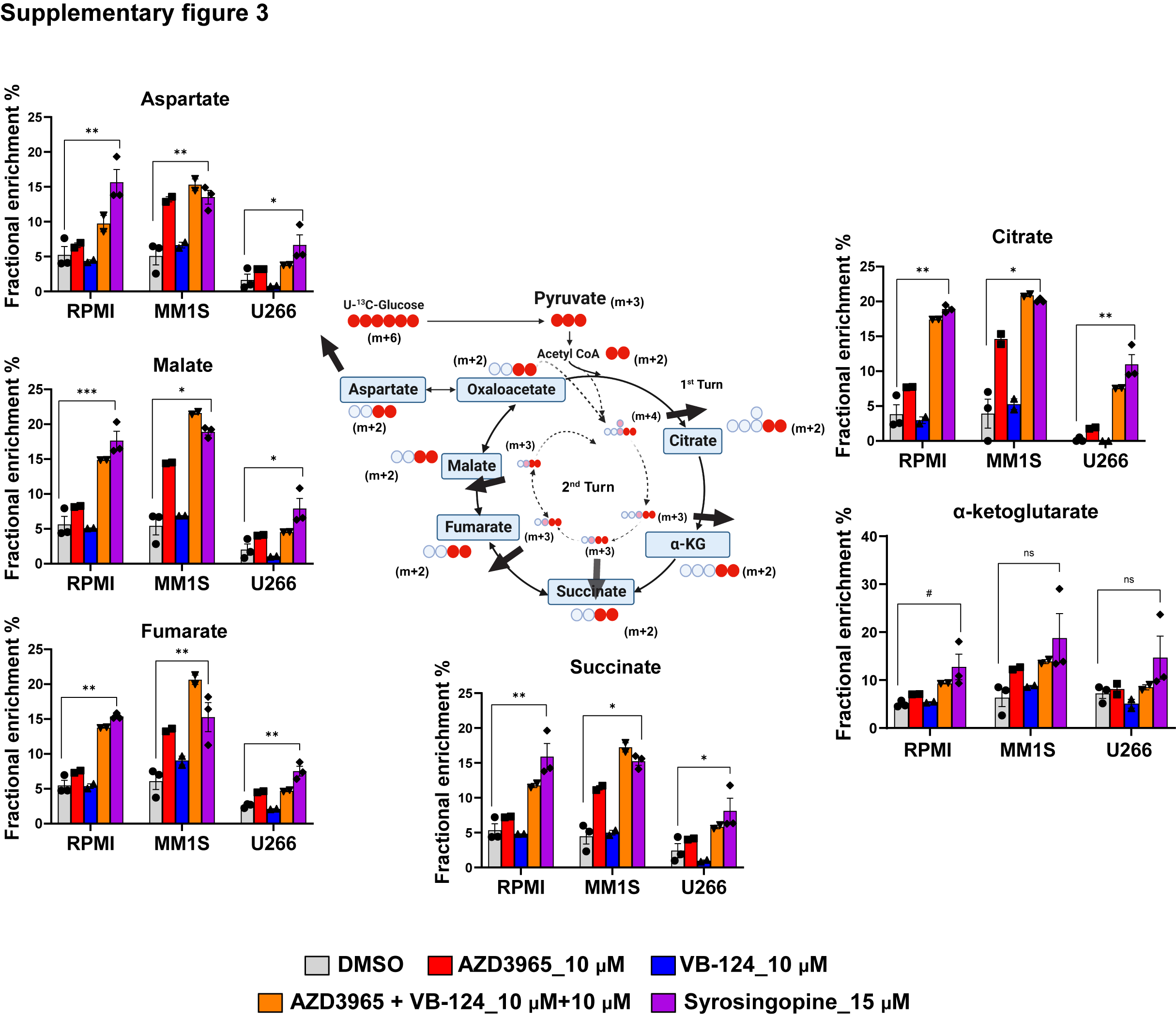

Supplement: Supplementary file 4 — Supplementary Fig. 3: Observed fractional enrichment of the isotopomers of the TCA cycle intermediates in HMCLs (RPMI-8226, MM1S and U266) upon the 2nd turn of the TCA cycle when cultured in RPMI-1640 cell culture media containing 100% enriched U- 13C-Glucose and treated with one of five different conditions: DMSO, AZD3965 10µM alone, VB124 10µM alone, combined AZD3965 10µM with VB124 10µM and syrosingopine 15µM. The entire experiment was repeated two times with all five conditions and the third replicate was conducted only with the DMSO and syrosingopine conditions. Error bars represent standard error of the mean (SEM). n.s. is non-significant, #p < 0.1, *p < 0.05, **p < 0.01, and ***p < 0.001 and ****p < 0.0001 by a paired t test [file 40170_2025_379_MOESM4_ESM.tif]

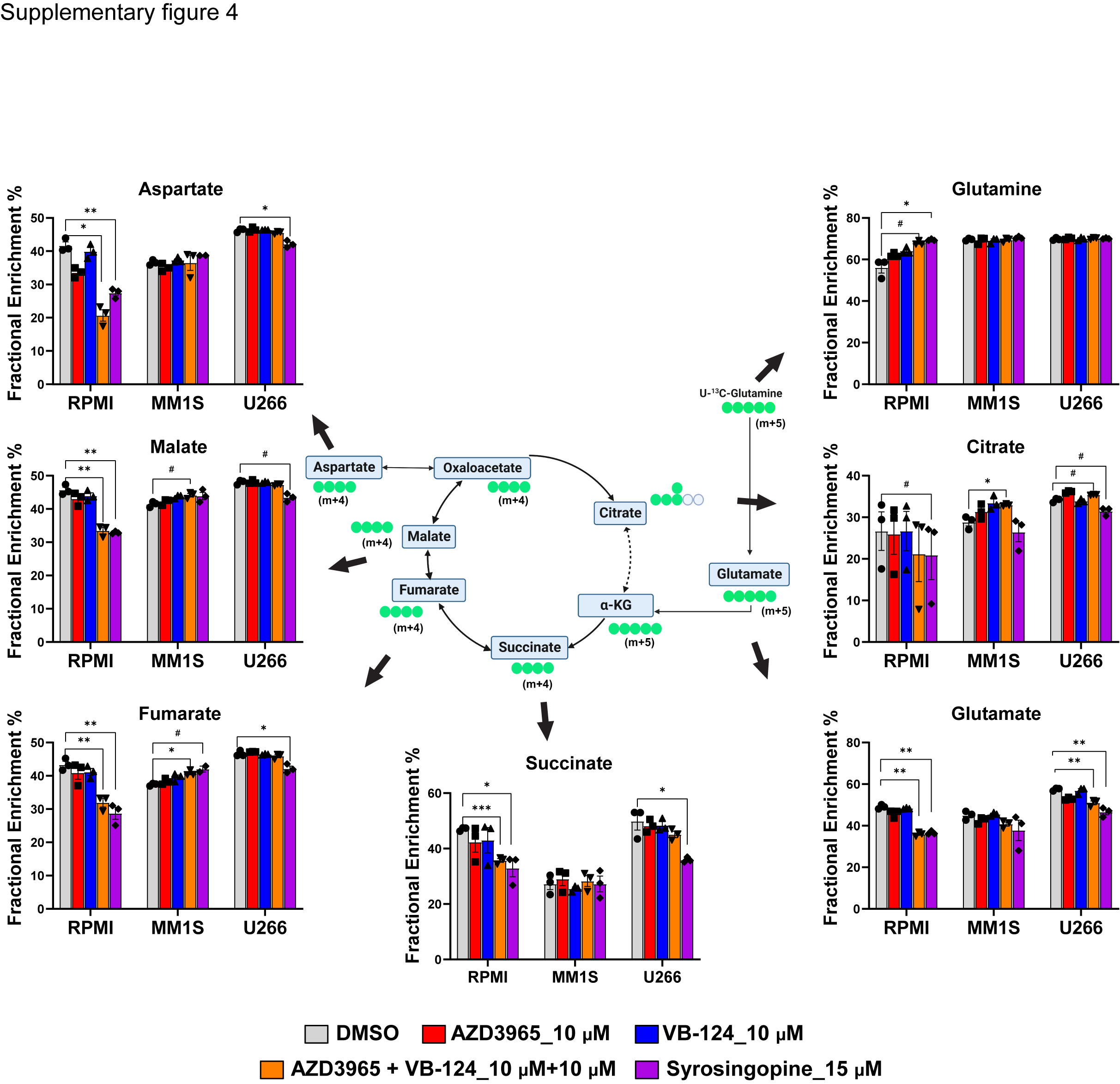

Supplement: Supplementary file 5 — Supplementary Fig. 4: Observed fractional enrichment of the isotopomers of the TCA cycle intermediates in HMCLs (RPMI-8226, MM1S and U266) cultured in RPMI-1640 cell culture media containing 2mM of 100% enriched U- 13C-Glutamine and treated with DMSO, AZD3965 10µM alone, VB124 10µM alone, combined AZD3965 10µM with VB124 10µM and syrosingopine 15µM. Error bars represent standard error of the mean (SEM). n.s. is non-significant, #p < 0.1, *p < 0.05, **p < 0.01, and ***p < 0.001 and ****p < 0.0001 by a paired t test [file 40170_2025_379_MOESM5_ESM.tif]

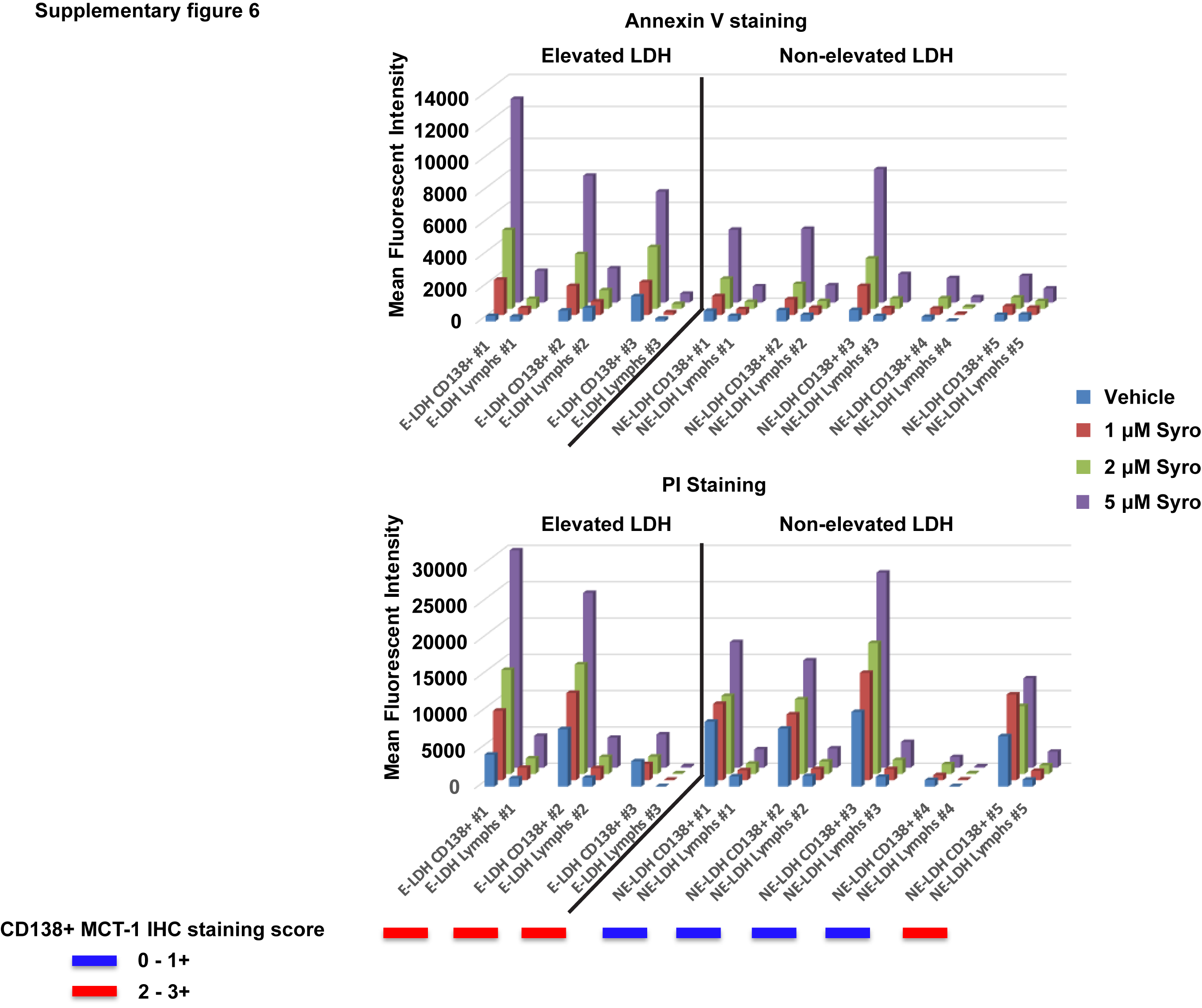

Supplement: Supplementary file 7 — Supplementary Fig. 6: Comparisons of PI and Annexin staining of primary CD138 + MM cells and their paired CD138- lymphocyte cells categorized by the level of their serum LDH at the time of sampling after after treatment with syrosingopine [file 40170_2025_379_MOESM7_ESM.tif]

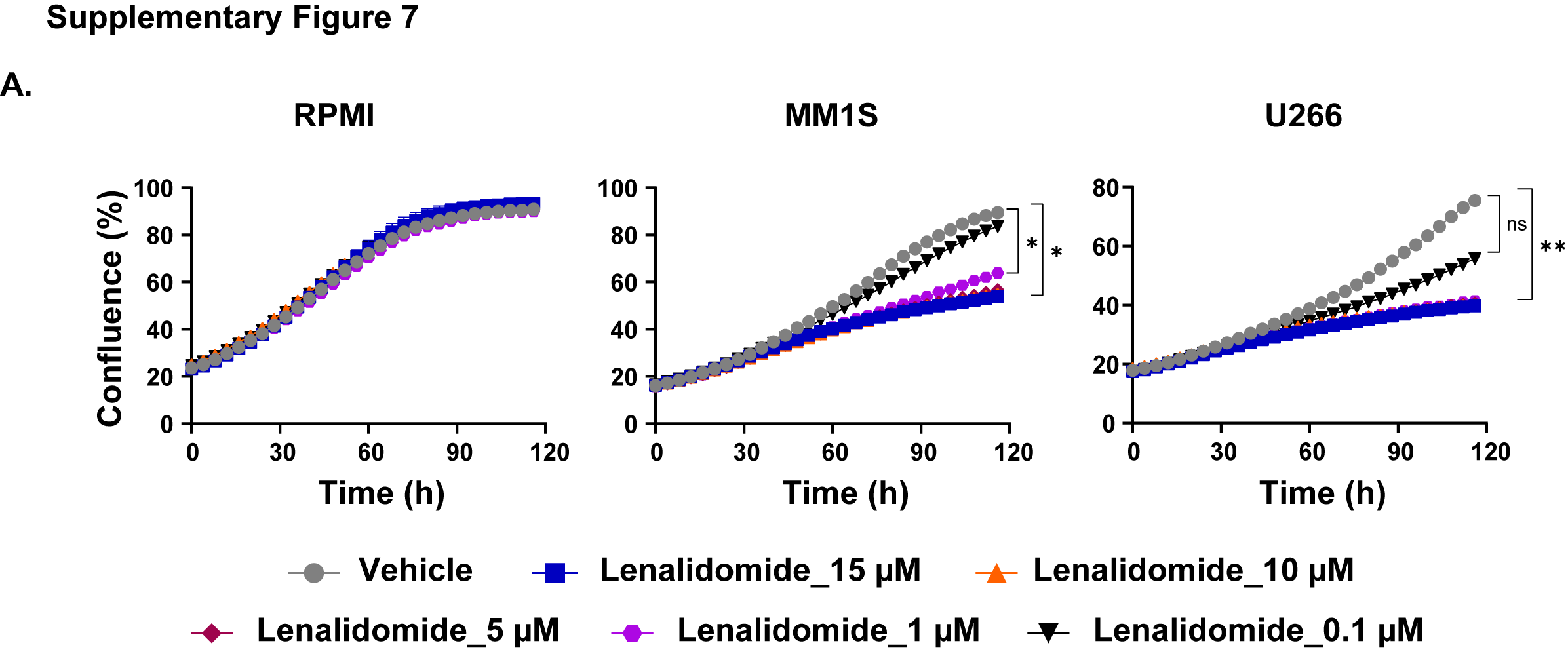

Supplement: Supplementary file 8 — Supplementary Fig. 7: Proliferation measured by confluency via live cell imaging in HMCLs (RPMI-8226, MM1S, and U266) treated with DMSO or different lenalidomide dose levels (0.1 µM, 1 µM, 5 µM, 10 µM and 15 µM). n.s. is non-significant, #p < 0.1, *p < 0.05, **p < 0.01, and ***p < 0.001 and ****p < 0.0001 by an independent t test [file 40170_2025_379_MOESM8_ESM.tif]
